# QUANTIFYING CONTRIBUTIONS OF WORK CHARACTERISTICS TO EDUCATIONAL DISPARITIES IN HEALTH-INDUCED WORK LIMITATIONS

**DOI:** 10.1093/geroni/igad104.1010

**Published:** 2023-12-21

**Authors:** Leah Abrams, Lisa Berkman

**Affiliations:** Tufts University, Medford, Massachusetts, United States; Harvard Center for Population and Development Studies, Harvard T.H. Chan School of Public Health, Cambridge, Massachusetts, United States

## Abstract

While public policies are incentivizing longer working lives, poor health and inhospitable working conditions pose challenges to working longer, especially for Americans with low educational attainment. This analysis sought to quantify how poor health and inhospitable working conditions each contribute to educational disparities in work disability in mid life and old age. Using the Health and Retirement Study (2006-2016), we showed that educational disparities in reporting “any impairment or health problem that limits the kind or amount of paid work” in ages 51-80 have been profound and persistent over time. Blinder-Oaxaca three-fold decomposition revealed that distributions of income and employer insurance made the largest contribution to explaining different rates of work limitations among respondents with versus without high school degrees, followed by work characteristics (physical job demands, insufficient hours) and health conditions (diabetes, lung disease). Comparing respondents with high school versus college degrees, distributions of health conditions mattered most (high blood pressure, lung disease, heart disease, stroke), followed by health behaviors (smoking, drinking). Health-induced work limitations are often used as a health outcome, but we found that work characteristics explained 57% of the disadvantage of those without a high school degree and 44% of the disadvantage of high school compared to college graduates. Therefore, improving work environments holds promise for reducing educational disparities in mid-to-late-life disability.

